# Global research trends on the interaction between gut microbiome and bile acids: a bibliometric and visualized analysis

**DOI:** 10.3389/fcimb.2025.1616995

**Published:** 2025-07-29

**Authors:** Fangli Luo, Luqiang Sun, Renhong Wan, Zhen Tian, Zhaoxuan He

**Affiliations:** ^1^ Department of Traditional Chinese Medicine, Affiliated Hospital of North Sichuan Medical College, Nanchong, China; ^2^ Clinical Medical College of Integrated Traditional Chinese and Western Medicine, North Sichuan Medical College, Nanchong, China; ^3^ College of Acupuncture and Tuina, Chengdu University of Traditional Chinese Medicine, Chengdu, China; ^4^ Department of Traditional Chinese Medicine, Chengdu Wenjiang District Maternal and Child Health Hospital of Traditional Chinese Medicine, Chengdu, China

**Keywords:** bibliometrics, gut microbiome, bile acids, Citespace, VOSviewer, visualization

## Abstract

**Background:**

An increasing number of studies have shown that gut microbiome-bile acids interactions play a crucial role in host health and disease. This bibliometric analysis aims to identify the global scientific output, research hotspots, and frontiers of gut microbiome-bile acids in the past two decades.

**Methods:**

We searched the relevant studies of gut microbiome-bile acids published between 2004 and 2024 in the Web of Science Core Collection database. Microsoft Excel 2019, VOSviewer 1.6.18, Tableau Desktop 2024.2.2, Scimago Graphica 1.0.45, and CiteSpace 6.2.R3 were used to analyze the publications, countries/regions, institutions, journals, authors, references, and keywords.

**Results:**

A total of 4795 original articles and reviews were collected. A visual analysis of the results showed that the number of publications increased rapidly over time. China published the most papers, the United States had the most citations, and the most productive institution was Shanghai Jiaotong University. The most prolific author was Jia Wei, and Jason M. Ridlon was the most frequently co-cited author. *Nutrients* was the most productive journal. In the keyword co-occurrence network, except for gut microbiome and bile acids, inflammation becomes the keyword with the highest frequency. Keywords and reference analysis show that metabolic diseases (such as obesity and type 2 diabetes mellitus), cancer (such as colorectal cancer), and disease-related mechanisms (such as tgr5 and pathway) are the hot topics and future research trends in this field.

**Conclusion:**

In this study, bibliometric analysis was utilized to explore the relationship between gut microbiome and bile acids. The findings can reflect the current hotspots and new directions of gut microbiome-bile acids, and provide an objective description and comprehensive guidance for future related studies.

## 1 Introduction

The gut microbiome is the largest and most complex micro-ecosystem in the human body, mainly composed of bacteria, fungi, germs, etc., with a total number of microorganisms up to several billion, known as the ‘invisible organ’ (Iacob et al., 2019; Luca et al., 2019; Li et al., 2020). Firmicutes and Bacteroidetes are the main parts of the gut microbiome, and they together maintain the health and stability of the gut (Behera et al., 2020). The number and proportion of gut microbiome may vary due to individual differences of the host, dietary habits, lifestyle, and other factors. The balance between the internal and external microbiome has an important impact on the host’s intestinal barrier function, energy metabolism, nutrient absorption, immune regulation, and other aspects (Backhed et al., 2005; Shi et al., 2023). When the gut microbiome is unbalanced, it can cause damage to the host mucosal barrier, immune system disorders, inflammatory response stimulation, metabolic product disorders, etc., leading to a variety of diseases (Finlay et al., 2020). In recent years, more and more studies have focused on the mechanism of gut microbiome affecting human health and disease, and related studies have shown that bile acids, as one of the important metabolites of gut microbiome, may play a key role in human-microbiome interactions (Lin et al., 2023).

Bile acids, a major component of bile, are synthesized from cholesterol in the liver and stored in the gallbladder ducts. They are secreted into the small intestine after eating to facilitate the digestion and absorption of triglycerides, cholesterol, and fat-soluble vitamins (Joyce and Gahan, 2016; Caliceti et al., 2022). Bile acids not only play a role in digestion but also act as signaling molecules to regulate host metabolism and immune response by activating downstream receptors (Shulpekova et al., 2022). Numerous studies have shown that bile acids are involved in the occurrence and development of diseases, and may be potential biomarkers and therapeutic targets for the diagnosis and prediction of various diseases (Brock et al., 2018; Bertolini et al., 2022; Long et al., 2023; Qi and Chen, 2023; Yin et al., 2023; Porru et al., 2024).

In recent years, increasing studies have shown that gut microbiome interacts and influences each other with bile acids (Wahlström et al., 2016). The gut microbiome can affect the synthesis, metabolism, and reabsorption of bile acids, while bile acids also have a significant impact on the composition, growth, and proliferation of the gut microbiome. Under normal conditions, this bidirectional regulatory balance maintains the stability of the gut microbiome and bile acids metabolism. If the balance between them is disrupted, it can lead to gut microbiome and bile acid disturbances, which affect the occurrence and progression of many diseases, including obesity, type 2 diabetes mellitus (T2DM), non-alcoholic fatty liver disease (NAFLD), inflammatory bowel disease (IBD), and tumors (Liu et al., 2018; Cai et al., 2022; Gao et al., 2022; Li et al., 2022, 2023).

At present, there are more and more studies in the field of gut microbiome-bile acids interactions (Collins et al., 2023). However, no study has systematically analyzed its research hotspots and global development trends. This gap makes it difficult for the academic community to grasp the overall picture of this field and predict the future direction. Therefore, simple and effective methods are needed to obtain the required information. Bibliometric analysis is a method to statistically evaluate the research status, research hotspots and development trends of the most influential research in a specific field (Ninkov et al., 2022; Donthu et al., 2021). Compared with traditional literature reviews, bibliometric analysis provides objective and statistically significant data for further analysis by researchers. In this study, bibliometric analysis was used to qualitatively and quantitatively analyze the different characteristics (including countries, institutions, journals, authors, and keywords) of publications in the field of gut microbiome-bile acids interactions research. It is helpful to comprehensively understand the cooperative network among countries, institutions, and researchers in this field, identify research hotspots, and predict future development trends, which may provide new ideas for further research, and provide guidance for basic and clinical research.

We obtained 4795 publications related to gut microbiome and bile acids from 2004 to 2024 in the Web of Science Core Collection (WOSCC). VOSviewer and CiteSpace software were used as bibliometric tools to analyze and visualize knowledge mappings. We aimed to comprehensively and systematically review the global state of research on gut microbiome and bile acids interactions over the period 2004–2024 and to fill the current gap of no bibliometric analysis in this area. In this study, comprehensive insights on publications, countries/regions, institutions, journals, authors, and keywords of the gut microbiome-bile acids interaction were obtained through bibliometrics to reveal the dynamic research trends in the gut microbiome-bile acids interaction and to highlight the current hotspots and frontiers of research and the future to further guide clinical and basic research.

## 2 Materials and methods

### 2.1 Data collection and retrieval strategies

We chose publications from the Web of Science Core Collection (WOSCC) dataset as our data source because the publications from the WOSCC dataset have higher quality and reputation and are highly recognized globally. The WOSCC data can make our research results more convincing, universal, and representative. Given the daily updates of this database, two researchers independently conducted a comprehensive search within a single day (August 22, 2024), utilizing subject headings in conjunction with free words. This approach was employed to mitigate any potential bias that could influence the results. The data retrieval formula was as follows: #1: TS = (‘Gastrointestinal Microbiomes’ OR ‘Microbiome, Gastrointestinal’ OR ‘Gastrointestinal Microbial Community’ OR ‘Gastrointestinal Microbial Communities’ OR ‘Microbial Community, Gastrointestinal’ OR ‘Gastrointestinal Microflora’ OR ‘Microflora, Gastrointestinal’ OR ‘Gastrointestinal Flora’ OR ‘Flora, Gastrointestinal’ OR ‘Gastrointestinal Microbiota’ OR ‘Gastrointestinal Microbiotas’ OR ‘Microbiota, Gastrointestinal’ OR ‘Gut Microbiome’ OR ‘Gut Microbiomes’ OR ‘Microbiome, Gut’ OR ‘Gut Microflora’ OR ‘Microflora, Gut’ OR ‘Gut Flora’ OR ‘Flora, Gut’ OR ‘Gut Microbiota’ OR ‘Gut Microbiotas’ OR ‘Microbiota, Gut’ OR ‘Intestinal Microbiome’ OR ‘Intestinal Microbiomes’ OR ‘Microbiome, Intestinal’ OR ‘Intestinal Flora’ OR ‘Flora, Intestinal’ OR ‘Intestinal Microbiota’ OR ‘Intestinal Microbiotas’ OR ‘Microbiota, Intestinal’ OR ‘Intestinal Microflora’ OR ‘Microflora, Intestinal’ OR ‘Enteric Bacteria’ OR ‘Bacteria, Enteric’ OR ‘Gastric Microbiome’ OR ‘Gastric Microbiomes’ OR ‘Microbiome, Gastric’); #2: TS = (‘Bile Acids’ OR ‘Acids, Bile’ OR ‘Bile Acid’ OR ‘Acid, Bile’); Final data: (#1 AND #2). Subsequently, a specific timespan was established, and only English-language documents published between January 1, 2004, and August 22, 2024, were included. The types of literature were restricted to original articles and reviews while excluding other types such as duplicate articles, editorials, letters, and meeting abstracts.

**Figure 1:**
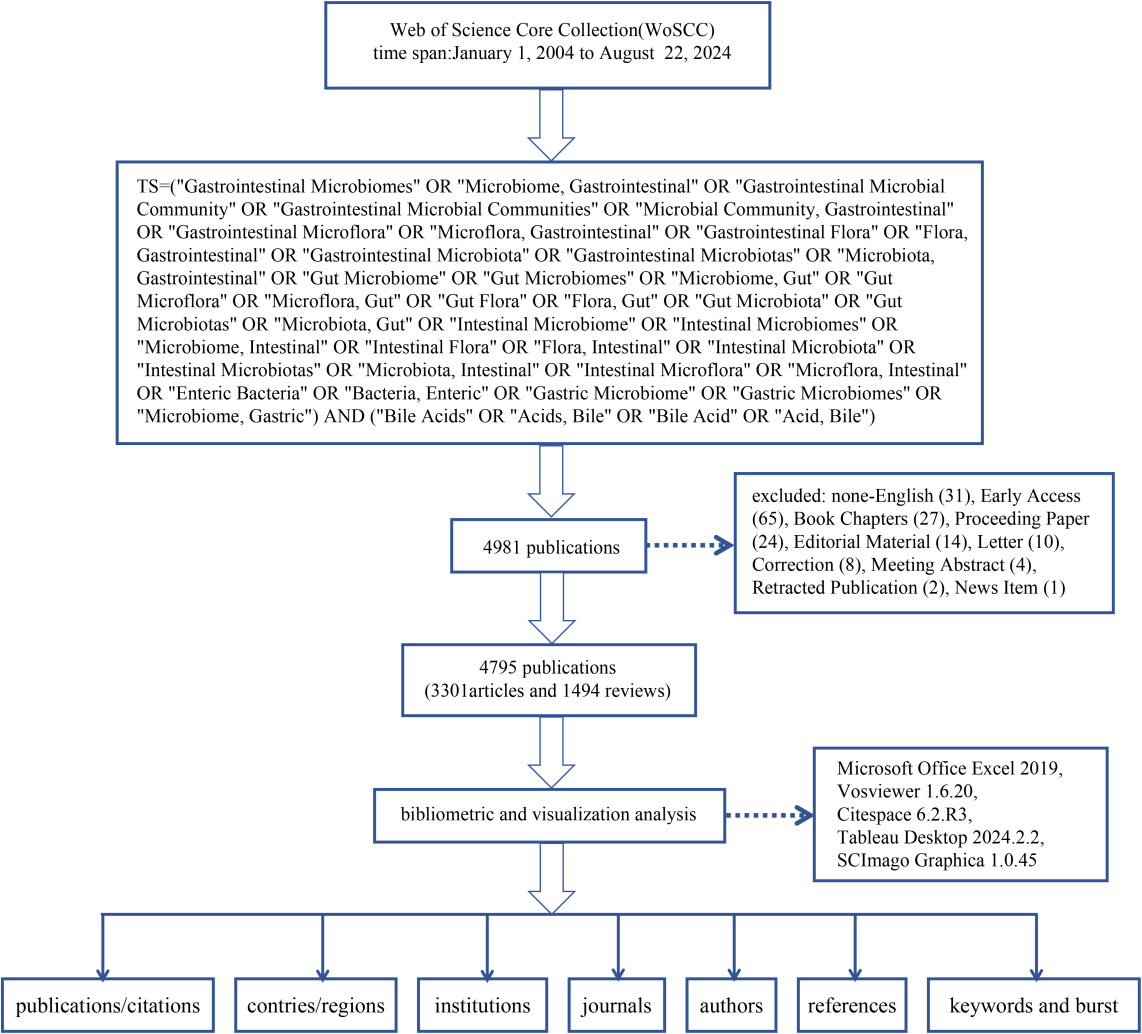
The flow chart of literature screening and research steps.

Two researchers performed the screening, and a third party was invited to assist in judgment if there was disagreement. After screening, a total of 4795 publications were included in this study. The exported data included ‘full record and citations’ and was in ‘plain text’ format. The detailed process of literature selection, screening, and analysis is shown in **Figure 1**.

### 2.2 Research software

The CiteSpace software developed by Professor Chen Chaomei can visualize and analyze the scientific research literature in a certain field, and then find the research hotspots and main research directions in this field (Synnestvedt et al., 2005). The VOSviewer software was jointly developed by Nees Jan van Eck and Ludo Waltman of the Science and Technology Research Center of Leiden University in the Netherlands. It is used to draw knowledge maps, which can visually analyze literature data such as keywords, authors, and countries (van Eck and Waltman, 2010). Based on CiteSpace and VOSviewer, two highly influential analysis tools, this study conducted a bibliometric analysis of the relevant literature on gut microbiome-bile acids interaction research published worldwide since 2004.

### 2.3 Statistical analysis and visualization

In this study, Microsoft Excel 2019 was used to draw the trend chart of the number of publications and citations, organize the data, and make related tables and bar charts. VOSviewer1.6.18 software was used to visualize the co-authorship analysis of institutions, countries/regions, and authors; the citation analysis of journals; the co-citation analysis of co-cited authors, co-cited journals and co-cited references; as well as the co-occurrence analysis of keywords. In terms of the layout of the maps, attraction is set to 2 and repulsion is set to -2, which can make nodes dispersed evenly and increase identification. At the same time, the cooperation maps of countries/regions were visualized using VOSviewer 1.6.18, Tableau Desktop 2024.2.2, and Scimago Graphica 1.0.45. CiteSpace 6.2.R3 was used for visual analysis of keyword clusters, timeline views, and keywords with strong citation bursts. CiteSpace 6.2.R3 parameters are as follows: time slice (2004-2024), years per slice (1), term source (entire selection), node type (keyword), selection criteria (top N = 50), g-index (k = 25), and pruning (pathfinder + pruning the merged network), and retain the default values for other parameters.

## 3 Results

### 3.1 Analysis of the number of publications and citations

**Figure 2:**
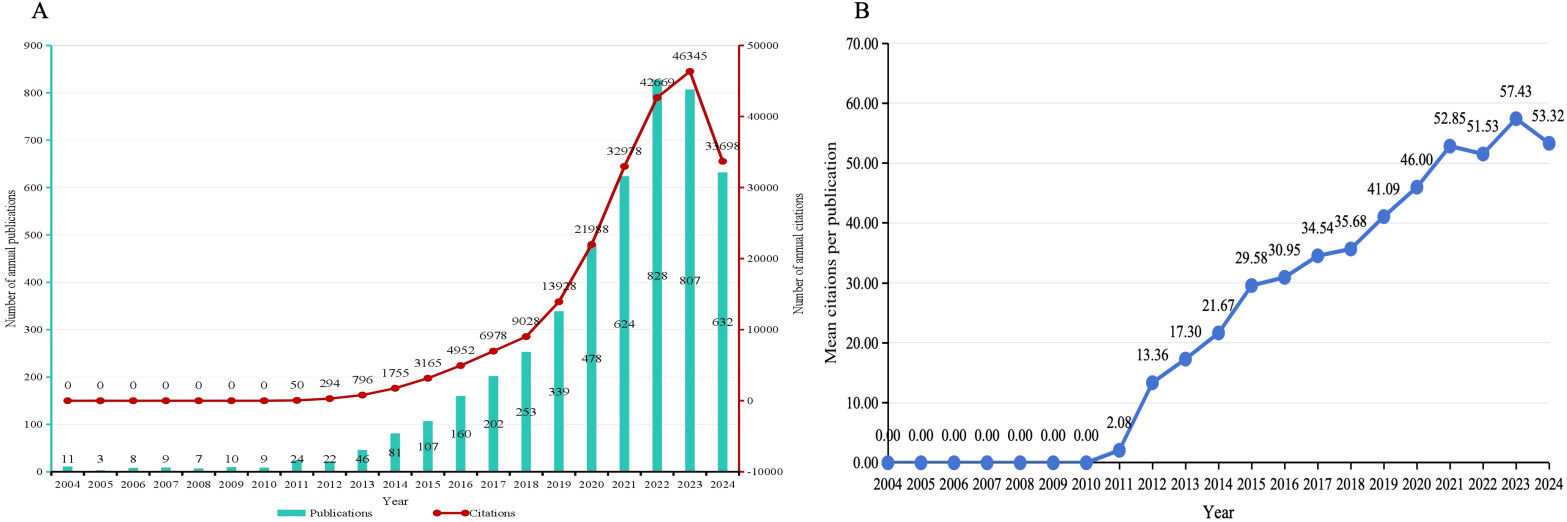
**(A)** Publication and citation timeline and trend of gut microbiome-bile acids interaction research from 2004 to 2024. The green bar chart shows the number of annual publications, and the red line shows citations, indicating an overall upward trend in both indicators over a given period. **(B)** The average number of citations per publication.

A total of 4795 papers were included in this study to count annual publications and citations. Due to the retrieval time, the number of publications published in 2024 was incomplete. As shown in **Figure 2A**, both the number of publications and the number of citations increased steadily, and the growth was faster after 2015. The number of publications peaked in 2022 (828 papers), and the number of citations peaked in 2023 (42669 papers). As shown in **Figure 2B**, the mean citations per publication increased year by year since 2011, with an overall obvious upward trend. Based on the data presented, it is evident that the field of gut microbiome-bile acids interaction has garnered significant attention from the academic community, as indicated by the substantial increase in both the number of publications and citations since 2015. This surge suggests a growing interest and expanding body of research, highlighting the importance and potential impact of this area of study. The consistent increase in mean citations per publication since 2011 further underscores the rising significance of these studies within the academic community.

### 3.2 Analysis of distributions of countries/regions

**Figure 3:**
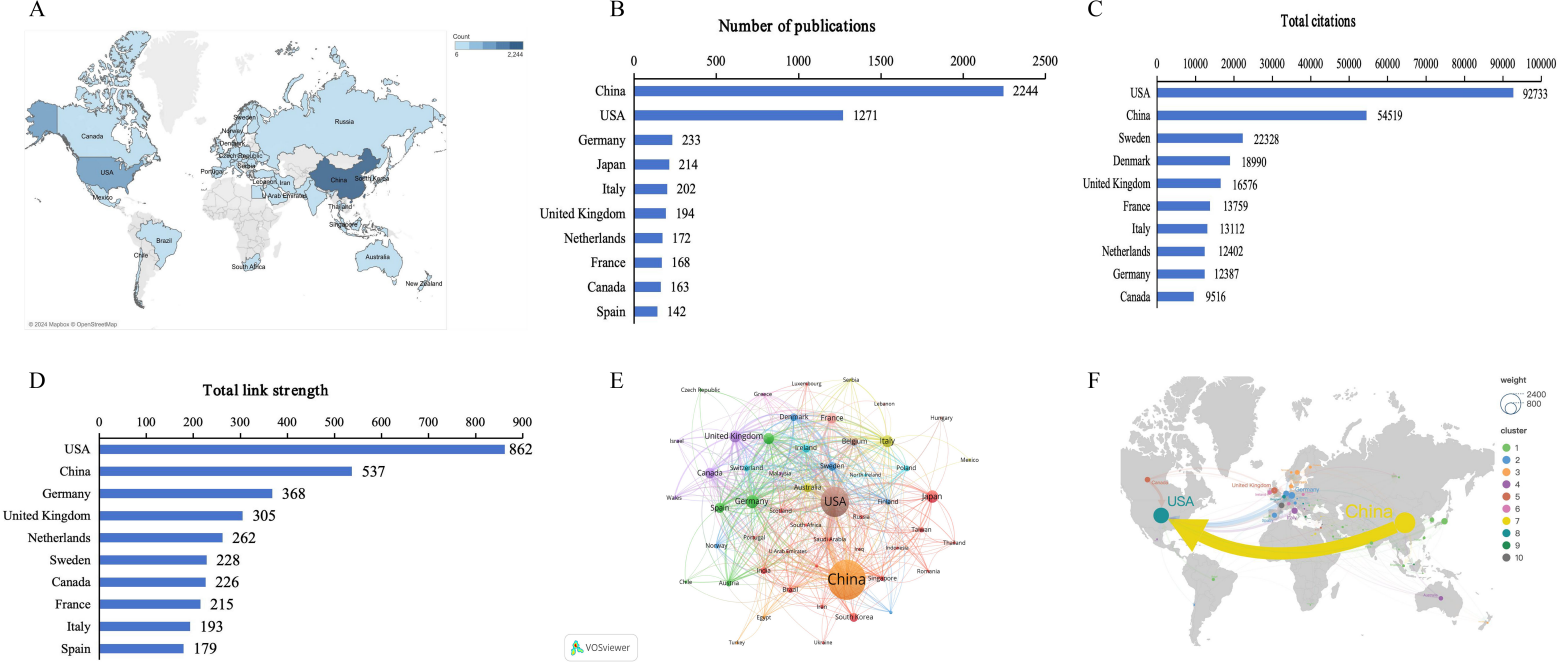
Countries/regions contributing to gut microbiome-bile acids interaction research. **(A)** World map of countries/regions’ distribution in this field. The darker the blue, the more the number of documents produced by the country. **(B)** The number of publications of the top 10 countries. **(C)** Total citations of related articles from the top 10 countries. **(D)**Total link strength of related articles from the top 10 countries. **(E)** Network map of countries’ co-authorship analysis with more than five publications. The size of nodes indicates the number of publications, and the larger the node, the more publications. The thickness of the lines indicates the strength of the relationship. **(F)** International collaboration and high-frequency collaboration countries/regions. The node represents the countries/regions, the node size represents the number of publications, and the lines between nodes represent the countries/regions cooperation.

Global contributions to research on gut microbiome-bile acids interaction were analyzed and represented by bibliometrics in a world map (**Figure 3A**). China contributed the greatest number of publications (2244, 46.80%), followed by the United States (1271, 26.51%), Germany (233, 4.86%), Japan (214, 4.46%), and Italy (202, 4.21%) (**Figure 3B**). Most of the publications were mainly published in Asian, North American, and European countries/regions. This distribution is closely related to the level of economic development of the above countries/regions and their respective emphasis on scientific research. Studies from the United States had the highest number of citations (92733 citations), followed by those from China (54519 citations), Sweden (22328 citations), Denmark (18990 citations), and the United Kingdom (16576 citations) (**Figure 3C**). The results of the above analysis reveal that China and the United States are leading contributors to the research on gut microbiome-bile acids interaction, reflecting their strong emphasis on scientific research. Additionally, the citation data suggests that while China has a high volume of publications, studies from the United States garner significantly more citations, indicating a possible difference in research impact.

The visual map of collaboration between different countries/regions shows that a total of 97 countries/regions contributed to publications in this field, including 53 countries with more than five publications in the field, which were analyzed in the co-authorship analysis (**Figure 3E**). The five countries with the highest total link strength were the United States (total link strength 862 times), China (537), Germany (368), the United Kingdom (305), and the Netherlands (202) (**Figure 3D**). Further analysis of international cooperation and high-frequency cooperation among countries/regions (**Figure 3F**) showed that the most frequent cooperation was from China to the United States (frequency = 255), then from Germany to the United States (65), the United Kingdom to the United States (47), Canada to the United States (46), and Italy to the United States (46). The collaboration in these countries/regions highlights that the United States is a central hub in the global gut microbiome-bile acids interaction research network, with strong collaborations with China, Germany, the United Kingdom, Canada, and Italy. This indicates a dynamic international collaboration landscape where the United States plays a pivotal role in fostering research partnerships. The high frequency of cooperation between China and the United States is particularly noteworthy, suggesting a synergistic relationship that likely accelerates advancements in this field.

### 3.3 Analysis of contributions of institutions

**Figure 4:**
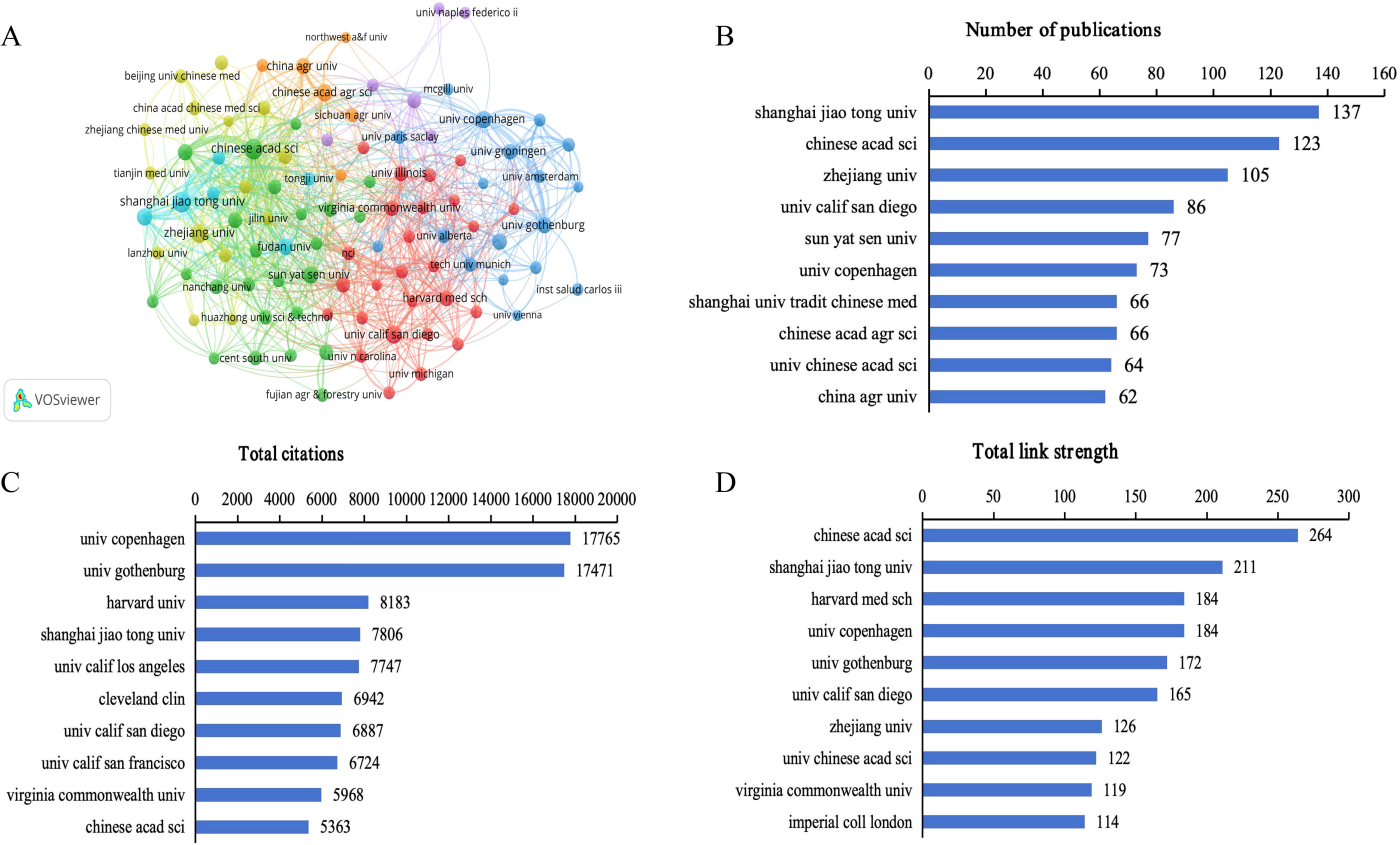
Analysis of institutions on gut microbiome-bile acids interaction research. **(A)** Network map of institutions’ co-authorship analysis with more than five publications. The size of nodes indicates the number of publications, and the larger the node, the more publications. The thickness of the lines indicates the strength of the relationship. **(B)** The number of publications of the top 10 institutions. **(C)** Total citations of the top 10 institutions. **(D)** Total link strength of the top 10 institutions.

Co-authorship analysis of institutions by VOSviewer shows the extensive collaboration between different institutions, with a total of 4514 institutions involved in research in this field, 544 of which had more than five publications (**Figure 4A**). Among the top 10 institutions in terms of the number of publications, eight were from China, one from the United States, and one from Denmark. Shanghai Jiaotong University contributed the greatest number of publications (137, 2.86%), followed by the Chinese Academy of Sciences (123, 2.57%), Zhejiang University (105, 2.19%), the University of California, San Diego (86, 1.79%), and Sun Yat-sen University (77, 1.61%) (**Figure 4B**). The five institutions with the highest total citations were the University of Copenhagen (17765), the University of Gothenburg (17471), Harvard Medical School (8183), Shanghai Jiaotong University (7806), and the University of California, Los Angeles (7747) (**Figure 4C**). The five institutions with the highest total link strength were the Chinese Academy of Sciences (264), Shanghai Jiaotong University (211), Harvard Medical School (184), the University of Copenhagen (184), and the University of Gothenburg (172) (**Figure 4D**). The co-authorship analysis underscores China’s prominence in the research landscape of gut microbiome-bile acids interaction, with institutions like Shanghai Jiaotong University and the Chinese Academy of Sciences leading in publication output. This dominance, coupled with strong domestic collaboration networks, highlights China’s strategic focus and substantial investment in this scientific domain. However, the high citation counts of institutions such as the University of Copenhagen and the University of Gothenburg suggest that while China excels in volume, European institutions may hold an edge in research impact and quality, indicating a potential area for China to enhance its global scientific standing through further innovation and international engagement.

### 3.4 Analysis of contributions of prolific authors and co-cited authors

**Figure 5:**
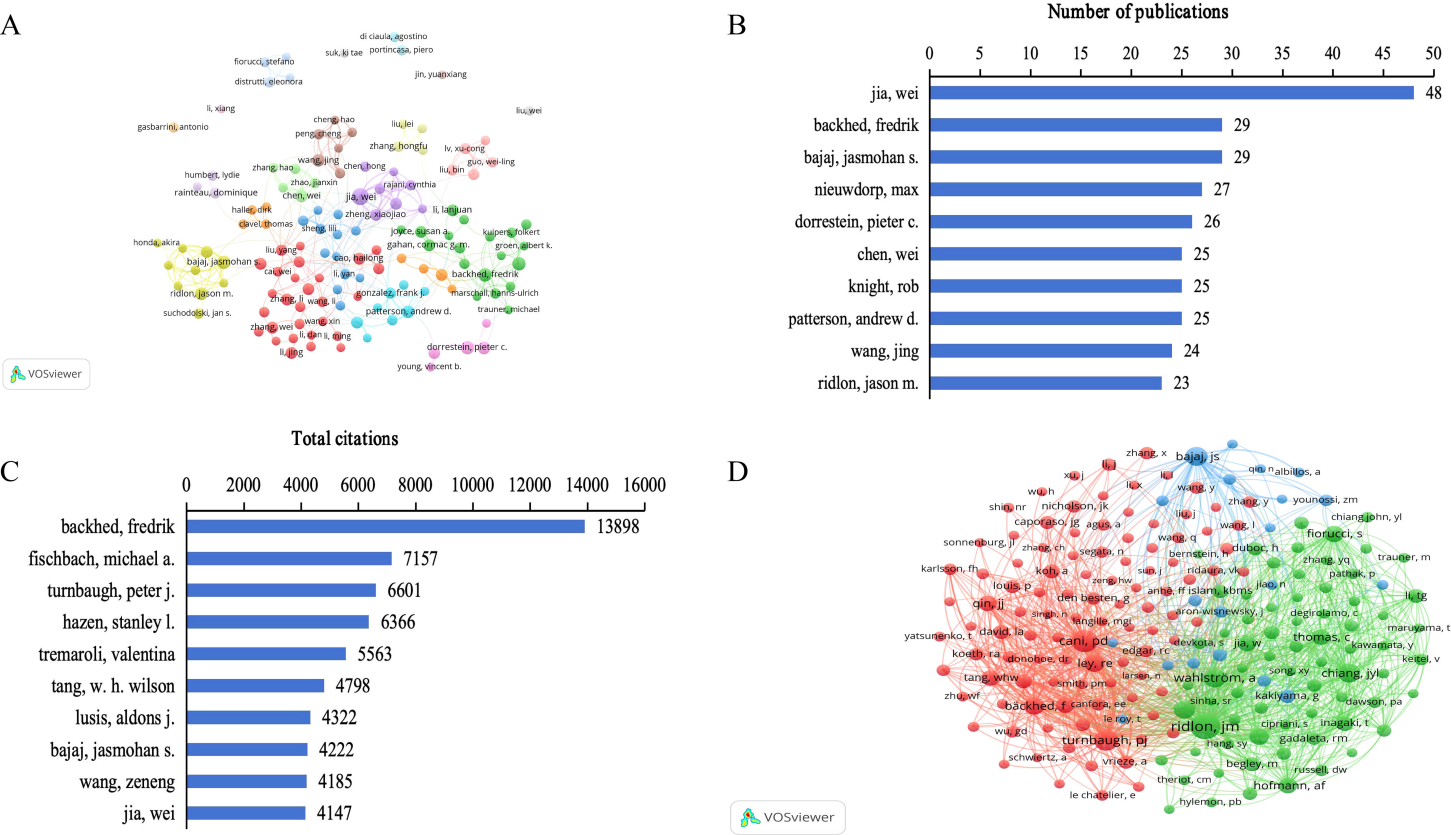
Analysis of authors on gut microbiome-bile acids interaction research. **(A)** Network map of authors’ co-authorship analysis with more than ten publications by VOSviewer. **(B)** Top 10 authors in the number of publications. **(C)** Top 10 authors in total citations. **(D)** Network map of co-cited authors’ co-citation analysis with more than 150 publications by VOSviewer.

The analysis of authors and co-cited authors can help identify core authors, understand the relationships among researchers, and reveal major collaborations between researchers in the field (**Figures 5A, D**). Node size is positively correlated with the number of publications or citations of a certain author, and the line thickness between nodes indicates the frequency of cooperation. The network maps of authors’ co-authorship and co-cited authors’ co-citation were visualized using the VOSViewer. There were 25596 authors, and 103136 co-cited authors in studies related to gut microbiome bile acids. According to Price Law (Price, 1963), the lower limit of core authors’ publications was calculated, and the formula was N=0.749n_max_1/2 (N is the lower limit of core authors’ publications, n_max_ represents the number of published publications by the authors with the most published publications), and the minimum number of core authors’ publications N≈5 was calculated, and only 641 qualified core authors were in the database (accounting for 2.5%). It indicates that the research in this field has formed a core group of authors with a certain scale. The most prolific and most-cited authors are summarized in **Table 1**. In terms of the number of publications, Jia Wei was the most productive author (48 articles), followed by Backhed Fredrik (29), Bajaj Jasmohan S. (29), Nieuwdorp Max (27), and Dorrestein Pieter c. (26) (**Figure 5B**). In terms of citations in this field, Ridlon Jason M. was ranked first (2051 citations), followed by Cani Patrice D. (1364), Turnbaugh Peter J. (1227), Wahlstrom A. (968), and Chiang John Y. L. (930) (**Figure 5C**). About the author, Rob Knight had the highest H-index (205), followed by Backhed Fredrik (105), Jia Wei (98), Bajaj Jasmohan S. (81), and Nieuwdorp Max (71) (**Table 1**). Concerning the co-cited author, the h-index of Cani Patrice D. (110) was ranked first, followed by Backhed Fredrik (105), Bajaj Jasmohan S. (81), Ley Ruth E. (77), and Fiorucci Stefano (68) (**Table 1**). The data on prolific and highly cited authors in the gut microbiome-bile acids field reveals a core group of researchers who have significantly influenced the direction and development of this area. Jia Wei’s high number of publications underscores a consistent and substantial contribution to the field’s body of knowledge. Ridlon Jason M.’s leading citation count highlights the profound impact of his work on the scientific community. Rob Knight’s highest h-index and Cani Patrice D.’s top co-citation h-index further emphasize their authoritative positions and the broad reach of their research. These findings suggest that these authors have not only been prolific in their output but have also driven significant advancements and collaborations, making them key figures in understanding and shaping the future of gut microbiome-bile acids interactions.

**Table 1:** The top 10 most productive authors and co-cited authors related to gut microbiome-bile acids.

Rank	Author	Country	Documents	Citations	TLS	H-index	Cited-author	Country	Citations	TLS	H-index
1	Jia Wei	China	48	4147	169	98	Ridlon, Jason M.	USA	2051	34970	33
2	Backhed, Fredrik	Sweden	29	13898	72	105	Cani Patrice D.	Belgium	1364	28184	110
3	Bajaj, Jasmohan S.	USA	29	4222	100	81	Turnbaugh, Peter J.	USA	1227	24260	2
4	Nieuwdorp, Max	Netherlands	27	2307	38	71	Wahlstrom, A.	Sweden	968	15765	22
5	Dorrestein, Pieter C.	USA	26	672	57	9	Chiang, John Y. L.	USA	930	16505	54
6	Chen, Wei	China	25	336	78	48	Bajaj, Jasmohan S.	USA	914	14863	81
7	Rob Knight	USA	25	1882	58	205	Sayin, Sama I.	Sweden	832	15652	5
8	Patterson, Andrew D.	USA	25	3535	80	64	Ley, Ruth E.	USA	803	16467	77
9	Jing Wang	China	24	496	27	26	Backhed, Fredrik	Sweden	742	15878	105
10	Ridlon, Jason M.	USA	23	3035	67	33	Fiorucci, Stefano	Italy	702	13360	68

### 3.5 Analysis of journals, co-cited journals, and research areas

**Figure 6:**
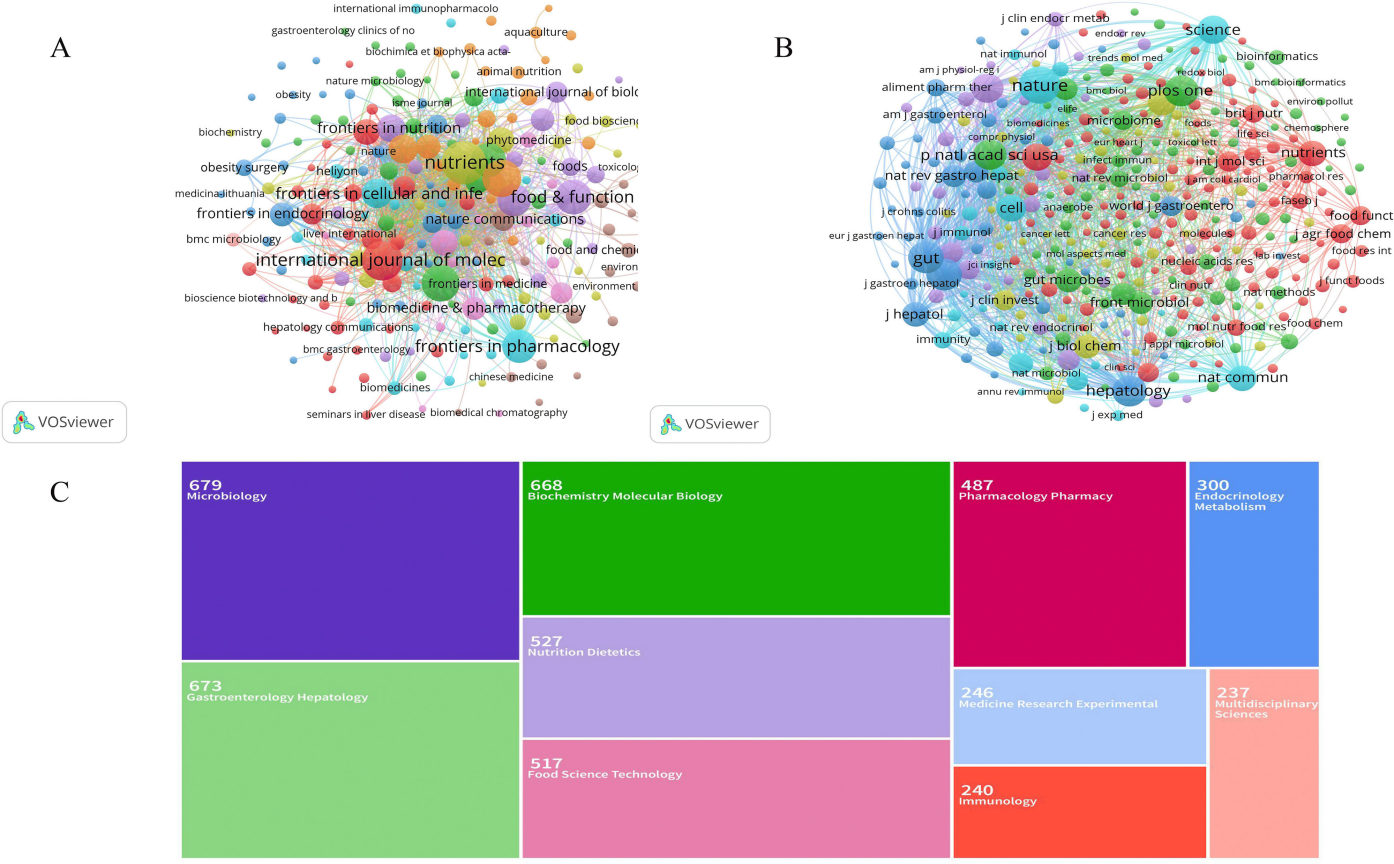
Analysis of journals on gut microbiome-bile acids interaction research. **(A)** Network map of journals’ co-cited analysis with more than 200 publications by VOSviewer. **(B)** Network map of journals’ co-cited analysis with more than 200 publications by VOSviewer. The node size represents the number of journal papers. The larger the node, the more journal papers. **(C)** Each color-coded section corresponds to a different field of study. The size of each section reflects the number of publications in the field of study, with Microbiology and Gastroenterology Hepatology being among the most studied areas.

Publications related to gut microbiome-bile acids included contributions from 966 citation journals and 13092 co-cited journals. We analyzed a total of 217 citation journals with more than five publications in the field (**Figure 6A**). **Table 2** shows the top 10 most popular journals for publishing publications on gut microbiome-bile acids. *Nutrients* (142 records, 2.96%) had the most publications, followed by the *International Journal of Molecular Sciences* (119, 2.48%), *Frontiers in Microbiology* (113, 2.36%), *Gut Microbes* (106, 2.21%), and *Food & Function* (97, 2.02%). Among these 10 journals, seven were included in the Journal Citation Reports (JCR) Q1, among which *Gut Microbes* had the highest impact factor (12.2). Six of the journals’ publishers are from Switzerland, two from the United States, and two from the United Kingdom. **Table 2** shows that, according to the h-index, the *Journal of Agricultural and Food Chemistry* (297) and *Scientific Reports* (213), are the two most influential journals. The preference for open-access journals in gut microbiome-bile acids research reflects a trend towards wider knowledge sharing, while the prominence of Swiss publishers highlights their significant influence in this academic field.

**Table 2:** Top 10 popular citation journals and cited journals.

Rank	Citation journals	Records (n)	Impact factor	JCR partition	H-index	Cited journals	Citations (n)	Impact factor	JCR partition	H-index
1	Nutrients	142	4.8	Q1	115	Nature	12349	50.5	Q1	1226
2	International Journal of Molecular Sciences	119	4.9	Q1	162	Gut	8499	23.0	Q1	293
3	Frontiers in Microbiology	113	4.0	Q2	135	Gastroenterology	8398	25.7	Q1	402
4	Gut Microbes	106	12.2	Q1	72	Plos One	7925	2.9	Q1	332
5	Food & Function	97	5.1	Q1	76	Hepatology	7673	12.9	Q1	361
6	Scientific Reports	88	3.8	Q1	213	Proceedings of the National Academy of Sciences of the United States of America	7658	9.4	Q1	771
7	Frontiers in Pharmacology	87	4.4	Q1	86	Cell Metabolism	6696	27.7	Q1	266
8	Journal of Agricultural and Food Chemistry	79	5.7	Q1	297	Science	6344	44.7	Q1	1186
9	Frontiers in Cellular and Infection Microbiology	68	4.6	Q2	75	Scientific Reports	5778	3.8	Q1	213
10	Metabolites	64	3.4	Q2	39	Cell	5243	45.5	Q1	776

We analyzed a total of 348 co-cited journals for all publications that were co-cited in more than 200 publications (**Figure 6B**). **Table 2** shows the top 10 cited journals that published related articles. The most cited journal was *Nature* (12349 citations), followed by *Gut* (8499), *Gastroenterology* (8398), *Plos One* (7925), and *Hepatology* (7673). According to the h-index, *Nature* (1226) and *Science* (1186) are the two most influential journals. The dominance of high-impact journals like *Nature* and *Science* in citations and h-index underscores their pivotal role in shaping research influence in this field, while also highlighting the diverse range of specialized journals contributing to its breadth.

Among the 4795 publications, the most representative research area was Microbiology (669 records, 13.95% of all publications), followed by Gastroenterology Hepatology (663, 13.83%), Biochemistry Molecular Biology (658, 13.72%), Nutrition Dietetics (517, 10.78%), and Food Science Technology (507, 10.57%) (**Figure 6C**, **Table 3**). The distribution of research areas in gut microbiome-bile acids reveals a strong interdisciplinary focus, with microbiology and gastroenterology-hepatology leading, while nutrition, biochemistry, and food science also play significant roles, reflecting the field’s complexity and collaborative nature.

**Table 3:** Top 10 well-represented research areas.

Rank	Research areas	Records (n)	% (of 4795)
1	Microbiology	669	13.95
2	Gastroenterology Hepatology	663	13.83
3	Biochemistry Molecular Biology	658	13.72
4	Nutrition Dietetics	517	10.78
5	Food Science Technology	507	10.57
6	Pharmacology Pharmacy	477	9.95
7	Endocrinology Metabolism	290	6.05
8	Medicine Research Experimental	236	5.13
9	Immunology	230	4.80
10	Multidisciplinary Sciences	227	4.73

### 3.6 Analysis of references and bursts detection

**Figure 7:**
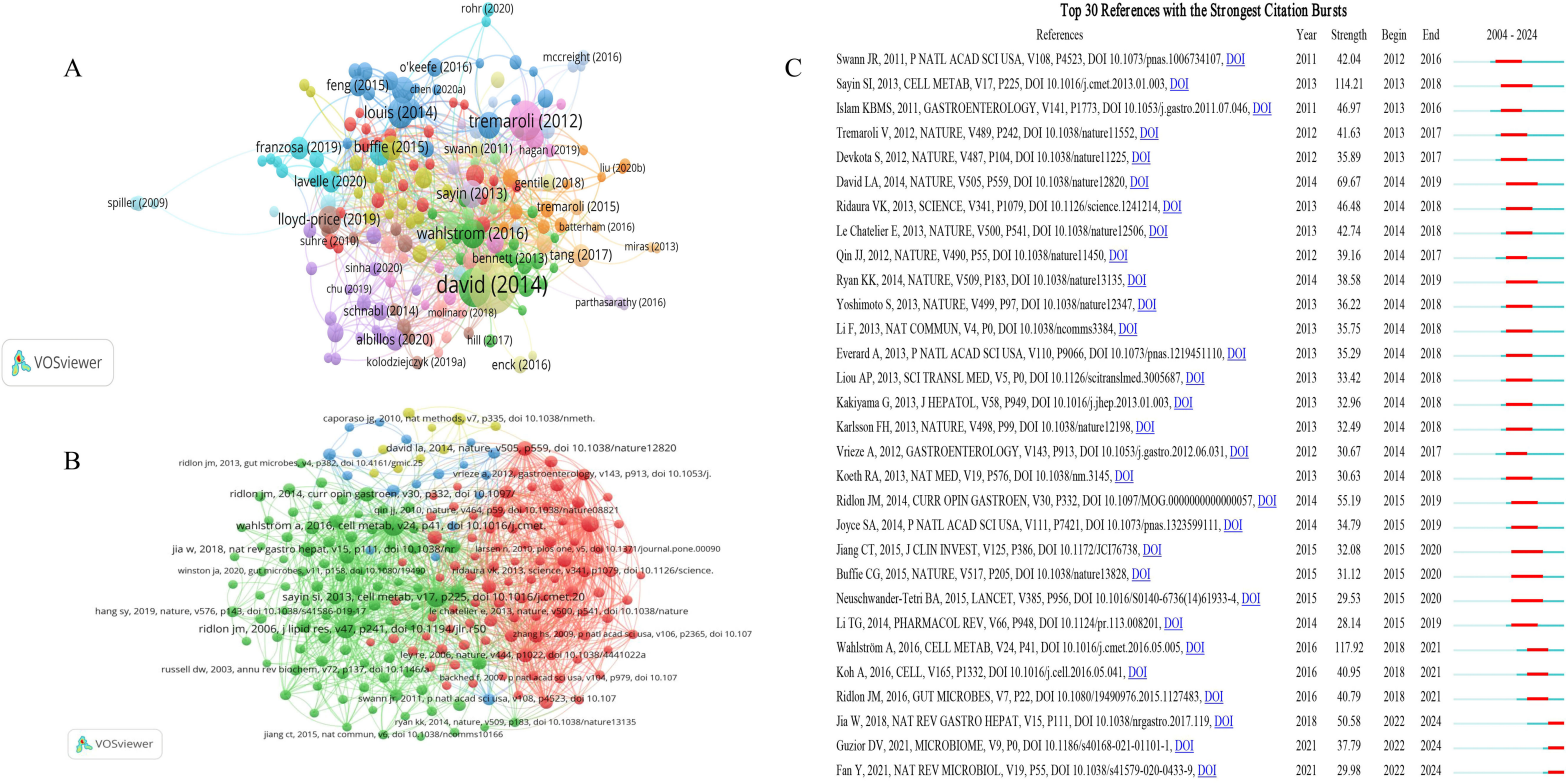
Analysis of references on gut microbiome-bile acids interaction. **(A)** Network map of references’ citation analysis with more than 200 citations. **(B)** Network map of references’ co-citation analysis with more than 100 citations. **(C)** The top 30 references with the strongest citation bursts on the gut microbiome and bile acids visualized by CiteSpace. These references are listed in order by the onset year of the citation burst, where the light blue line indicates that the keyword does not appear, the dark blue line indicates that the keyword begins to appear, and the red line indicates the duration of the citation burst.

The citation analysis showed that 198 documents had more than 200 citations (**Figure 7A**). **Table 4** lists the top ten documents with the highest citations. There were 6427 citations for ‘Diet rapidly and reproducibly alters the human gut microbiome’ (David et al., 2014), followed by ‘Functional interactions between the gut microbiota and host metabolism’ (Tremaroli and Bäckhed, 2012), with 3130 citations. The third-ranked article with the largest number of citations was ‘Intestinal microbiota metabolism of L-carnitine, a nutrient in red meat, promotes atherosclerosis’ (Koeth et al., 2013), with 3016 citations. The citation analysis highlights seminal works that have significantly shaped the field of gut microbiome research. These high-citation articles underscore their importance in understanding the dynamic relationship between diet, microbiome, and host health. These studies have not only garnered substantial attention but also driven further exploration into the metabolic and health implications of gut microbiome interactions.

**Table 4:** The top 10 gut microbiome-bile acids related articles with the most citations.

Title	Country	Date of publishing	Citations	Total link strength	Journal	JCR
Diet rapidly and reproducibly alters the human gut microbiome	USA	2014	6427	35	Nature	Q1
Functional interactions between the gut microbiota and host metabolism	Sweden	2012	3130	18	Nature	Q1
Intestinal microbiota metabolism of L-carnitine, a nutrient in red meat, promotes atherosclerosis	USA	2013	3016	22	Nature Medicine	Q1
The gut microbiota, bacterial metabolites and colorectal cancer	United Kingdom	2014	1843	15	Nature Reviews Microbiology	Q1
Intestinal Crosstalk between Bile Acids and Microbiota and Its Impact on Host Metabolism	Denmark	2016	1648	44	Cell Metab	Q1
Gut microbiota regulates bile acid metabolism by reducing the levels of tauro-beta-muricholic acid, a naturally occurring FXR antagonist	Sweden	2013	1566	68	Cell Metab	Q1
Multi-omics of the gut microbial ecosystem in inflammatory bowel diseases	USA	2019	1476	8	Nature	Q1
Gut microbiota functions: metabolism of nutrients and other food components	United Kingdom	2018	1454	4	European Journal of Nutrition	Q2
The Impact of Dietary Fiber on Gut Microbiota in Host Health and Disease	Sweden	2018	1382	15	Cell Host Microbe	Q1
Precision microbiome reconstitution restores bile acid mediated resistance to Clostridium difficile	USA	2015	1244	18	Nature	Q1

Co-cited references refer to documents that have been co-cited by the 4795 studies included in the analysis. References with citation burst refer to documents that have been highly cited in a period of time. We analyzed 211 references that were co-cited in more than 100 citations (**Figure 7B**). The top 10 co-cited references are listed in **Table 5**. These highly co-cited studies were all reviews. All of the co-cited references focused on the complex interaction between bile acids and the gut microbiome, and the potential impact of this interaction on host metabolism and health. Among them, the paper entitled ‘Intestinal Crosstalk between Bile Acids and Microbiota and Its Impact on Host Metabolism’ has received the most number of co-citations (n=893) (Wahlström et al., 2016). The paper entitled ‘Gut microbiota regulates bile acid metabolism by reducing the levels of tauro-beta-muricholic acid, a naturally occurring FXR antagonist’ ranked second (n=832) (Sayin et al., 2013). Both of the two articles were published on Cell Metab.

**Table 5:** Top 10 co-cited references related to gut microbiome-bile acids interaction.

Rank	Co-cited reference	Co-citation
1	Wahlström A, Sayin SI, Marschall HU, Bäckhed F. Intestinal Crosstalk between Bile Acids and Microbiota and Its Impact on Host Metabolism. Cell Metab. 2016 Jul 12;24(1):41-50.	893
2	Sayin SI, Wahlström A, Felin J, Jäntti S, Marschall HU, Bamberg K, Angelin B, Hyötyläinen T, Orešič M, Bäckhed F. Gut microbiota regulates bile acid metabolism by reducing the levels of tauro-beta-muricholic acid, a naturally occurring FXR antagonist. Cell Metab. 2013 Feb 5;17(2):225-35.	832
3	Ridlon JM, Kang DJ, Hylemon PB. Bile salt biotransformations by human intestinal bacteria. J Lipid Res. 2006 Feb;47(2):241-59.	779
4	Turnbaugh PJ, Ley RE, Mahowald MA, Magrini V, Mardis ER, Gordon JI. An obesity-associated gut microbiome with increased capacity for energy harvest. Nature. 2006 Dec 21;444(7122):1027-31.	556
5	Jia W, Xie G, Jia W. Bile acid-microbiota crosstalk in gastrointestinal inflammation and carcinogenesis. Nat Rev Gastroenterol Hepatol. 2018 Feb;15(2):111-128.	458
6	Ridlon JM, Kang DJ, Hylemon PB, Bajaj JS. Bile acids and the gut microbiome. Curr Opin Gastroenterol. 2014 May;30(3):332-8.	432
7	David LA, Maurice CF, Carmody RN, Gootenberg DB, Button JE, Wolfe BE, Ling AV, Devlin AS, Varma Y, Fischbach MA, Biddinger SB, Dutton RJ, Turnbaugh PJ. Diet rapidly and reproducibly alters the human gut microbiome. Nature. 2014 Jan 23;505(7484):559-63.	410
8	Islam KB, Fukiya S, Hagio M, Fujii N, Ishizuka S, Ooka T, Ogura Y, Hayashi T, Yokota A. Bile acid is a host factor that regulates the composition of the cecal microbiota in rats. Gastroenterology. 2011 Nov;141(5):1773-81.	392
9	Ley RE, Turnbaugh PJ, Klein S, Gordon JI. Microbial ecology: human gut microbes associated with obesity. Nature. 2006 Dec 21;444(7122):1022-3.	365
10	Watanabe M, Houten SM, Mataki C, Christoffolete MA, Kim BW, Sato H, Messaddeq N, Harney JW, Ezaki O, Kodama T, Schoonjans K, Bianco AC, Auwerx J. Bile acids induce energy expenditure by promoting intracellular thyroid hormone activation. Nature. 2006 Jan 26;439(7075):484-9.	354

References with citation burst refer to documents that have been highly cited in a period of time. As shown in **Figure 7C**, the threshold was set to the top 30 in a 1-year slice in CiteSpace, and strong citation bursts with a minimum duration of three years were found in 30 co-cited references. The reference with the strongest burst strength (strength = 117.92, burst period = 2018–2021) is ‘Intestinal Crosstalk between Bile Acids and Microbiota and Its Impact on Host Metabolism’ published by Wahlström A et al (Wahlström et al., 2016), same with the most co-cited reference. The identification of Wahlström et al.’s work as having the strongest citation burst underscores the rapid and significant impact this research has had on the field. This indicates that the study has not only been highly influential but also has catalyzed further investigation into the metabolic interactions between bile acids and the gut microbiota, highlighting its pivotal role in shaping contemporary research directions.

### 3.7 Analysis of keywords

**Figure 8:**
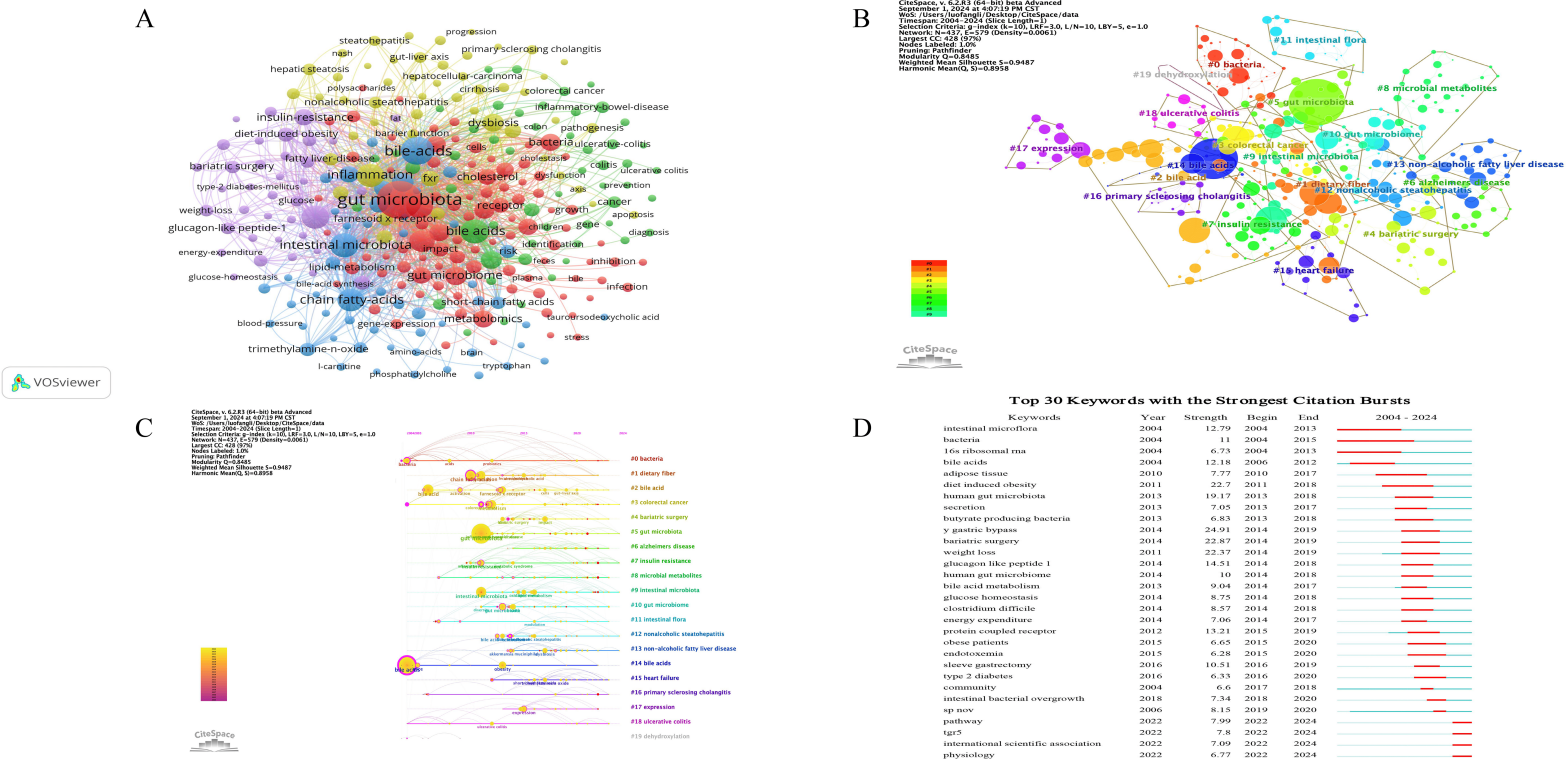
Analysis of keywords on gut microbiome-bile acids interaction. **(A)** The visualization map of the keywords. Nodes represent keywords; the larger the font, the more frequency; different colors represent different clusters; and the connection and thickness between nodes represent the connection and closeness between keywords. **(B)** The visualization map of the cluster analysis for keywords. Different colors represent different clusters; The smaller the value of the cluster label, the larger the cluster scale. **(C)** The timeline view of the cluster analysis for keywords. The X-axis of the timeline view is the year of occurrence of keywords in the cluster, and the Y-axis is the cluster of keywords, which can further show the occurrence, end, and time trend of each cluster and reflect the importance and distribution time span of a certain cluster. **(D)** The top 30 keywords with the strongest citation bursts on the gut microbiome and bile acids visualized by CiteSpace. These keywords are listed in order by the onset year of the citation burst, where the light blue line indicates that the keyword does not appear, the dark blue line indicates that the keyword begins to appear, and the red line indicates the duration of the citation burst.

Keywords highly summarize the core views and themes of the included literature, which can reflect the hot spots and frontiers of the field. According to the co-occurrence analysis of keywords by VOSviewer, there are a total of 12415 keyword nodes and 286 keywords with frequency ≥30 times, as shown in **Figure 8A**. The five keywords with the highest frequency were ‘gut microbiota’ (2425 occurrences), ‘bile-acids’ (1131), ‘bile acids’ (782), ‘intestinal microbiota’ (770), and ‘inflammation’ (747). **Table 6** lists the top twenty keywords with the highest frequency. By summarizing them, it can be found that the high-frequency keywords mainly focus on the gut microbiome, bile acid metabolism and related metabolites, and the role of the gut microbiome and bile acids in pathological states of diseases (such as inflammation, obesity, and insulin resistance). The co-occurrence analysis of keywords reveals that the field of gut microbiome-bile acids interaction is highly focused on understanding the interplay between gut microbiota and bile acid metabolism, as well as their roles in various pathological states such as inflammation, obesity, and insulin resistance. The high frequency of keywords like ‘gut microbiota’ and ‘bile acids’ indicates that these topics are central to current research, reflecting the field’s interest in elucidating the mechanisms and implications of this interaction for human health and disease.

**Table 6:** Top 20 occurrence analysis of keywords on gut microbiome-bile acids interaction research.

Rank	Keyword	Frequency	Rank	Keyword	Frequency
1	gut microbiota	2425	11	gut microbiome	402
2	bile-acids	1131	12	microbiome	398
3	bile acids	782	13	dysbiosis	368
4	intestinal microbiota	770	14	metabolomics	359
5	inflammation	747	15	bile acid	348
6	obesity	726	16	bacteria	334
7	chain fatty-acids	688	17	disease	333
8	metabolism	675	18	expression	308
9	microbiota	598	19	insulin-resistance	302
10	bile-acid	403	20	health	298

To better study and show the research hotspots in this field, the obtained keywords were cluster analyzed and the keywords cluster map was drawn. The log-likelihood ratio (LLR) algorithm in CiteSpace was used for cluster analysis of literature keywords, K=10 was set, pathfinder and pruning of the merged networks were selected. The software provides a modularity value (Q value) and a weighted mean silhouette value (S value) for evaluating map structure and cluster clarity (Yang et al., 2022). Generally, Q > 0.3 indicates that the cluster structure is significant. S > 0.7 indicates that the clustering results are convincing. The clustering map of 437 nodes and 3907 connections was analyzed in **Figure 8B**, and a total of 20 effective clusters were formed. The Q value was 0.8485, and the S value was 0.9487, which indicated that the network had high homogeneity, close connections between keywords, reasonable cluster structure, and high credibility. Using CiteSpace, we categorized these keyword clusters into 20 categories, including #0 ‘bacteria’, #1 ‘dietary fiber’, #2 ‘bile acid’, #3 ‘colorectal cancer’, #4 ‘bariatric surgery’, #5 ‘gut microbiota’, #6 ‘alzheimers disease’, #7 ‘insulin resistance’, #8 ‘microbial metabolites’, #9 ‘intestinal microbiota’, #10 ‘gut microbiome’, #11 ‘intestinal flora’, #12 ‘nonalcoholic steatohepatitis’, #13 ‘non-alcoholic fatty liver disease’, #14 ‘bile acids’, #15 ‘heart failure’, #16 ‘primary sclerosing cholangitis’, #17 ‘expression’, #18 ‘ulcerative colitis’, and #19 ‘dehydroxylation’. (**Figure 8B**). From the cluster names and their sub-clusters, #3, #6, #7, #12, #13, #15, #16, #18 focused on diseases, #0, #5, #8, #9, #10, #11 focused on microbiome and its metabolism, #14 focused on bile acids, and #17 and #19 focused on mechanisms. The keyword cluster analysis reveals a well-defined and highly credible network of research hotspots in the field of gut microbiome-bile acids interaction. The high modularity (Q value) and silhouette (S value) scores indicate robust and distinct clusters, highlighting key areas such as specific diseases (#3, #6, #7, #12, #13, #15, #16, #18), microbiome and its metabolism (#0, #5, #8, #9, #10, #11), bile acids (#14), and underlying mechanisms (#17, #19). This clustering underscores the field’s focus on both mechanistic understanding and clinical applications, demonstrating a balanced research approach that integrates fundamental science with potential therapeutic targets.

The timeline view of the cluster analysis for keywords can directly reflect the dynamic research changes of gut microbiome-bile acids. Timeline view analysis was performed based on keyword co-occurrence, as shown in **Figure 8C**. The keywords cluster themes #0, #3, #14, and #18 appeared earlier, while #3, #4, #5, #6, #7, #12, #13, #15, and #16 have continued to the present. The timeline of keyword cluster analysis shows that early research on the interaction between the gut microbiome and bile acids was based on general microbiome and bile acids interaction, but in recent years, the research has mainly focused on related diseases. Such as colorectal cancer, Alzheimer’s disease, insulin resistance, non-alcoholic steatohepatitis, non-alcoholic fatty liver disease, heart failure, primary sclerosing cholangitis, ulcerative colitis, the interaction between intestinal flora and bile acids, etc. These results reveal a shift in research focus from basic microbiome-bile acids interactions to their role in metabolic and systemic diseases, highlight the increasing emphasis on translational applications and mechanistic studies in recent years, and indicate the progress in this field from mechanistic studies to therapeutic applications.

The burst analysis of keywords can show the transfer of research hotspots in different periods, and reveal the potential development trend and frontier research. The higher the strength of keyword citation burst, the greater the influence. **Figure 8D** presents the top 30 keywords with the strongest citation bursts. Notably, ‘y gastric bypass’ (24.91), ‘bariatric surgery’ (22.87), ‘diet induced obesity’ (22.7), and ‘weight loss’ (22.37) emerged as the keywords with the most strength citation bursts, indicating that diet induced obesity was a key research disease in this field. The keywords ‘y gastric bypass’, ‘bariatric surgery’, and ‘weight loss’ were all the treatments for obesity. Over time, the keywords ‘intestinal microflora’ (burst duration from 2004 to 2013, 9 years), ‘16s ribosomal rna’ (2004-2013, 9 years), and ‘bacteria’ (2004-2015, 11 years) experienced the most persistent attention. In addition, keywords such as ‘intestinal bacterial overgrowth’ (burst duration from 2018 to 2020), ‘sp nov’ (2018-2020), ‘pathway’ (2022-2024), ‘tgr5’ (2022-2024), and ‘international scientific association’ (2022-2024) were attractive more recently, revealing that these keywords represented the popular research topics in recent years and even in recent years. The burst analysis of keywords highlights a significant shift in research focus within the gut microbiome-bile acids field, from foundational microbiological studies to more recent emphasis on obesity-related interventions and emerging mechanistic pathways. The strong citation bursts for terms like ‘y gastric bypass’ and ‘bariatric surgery’ indicate a growing interest in surgical treatments for obesity, reflecting the field’s practical turn towards addressing prevalent health issues. Meanwhile, the persistent attention to keywords such as ‘intestinal microflora’ and ‘16s ribosomal rna’ underscores the enduring importance of microbiome characterization. The recent emergence of keywords like ‘pathway’ and ‘tgr5’ suggests an ongoing exploration of specific metabolic and signaling pathways, indicating the field’s progression towards more detailed mechanistic understandings and potential therapeutic targets.

## 4 Discussion

### 4.1 General information on gut microbiome-bile acids research

In this study, bibliometric analysis and network visualization techniques were used to provide a comprehensive review of the research progress on the interaction between gut microbiota and bile acids, identify hot topics in the field, and predict potential directions for future research. We retrieved 4795 original articles and reviews published from 2004 to 2024.

Global variation in the number of publications and citations can reflect the research speed and the developmental trends in scientific research fields (Perez-Riverol et al., 2022). Over the past two decades, the number of annual publications has been on an upward trend, which can be divided into two phases of slow and rapid growth. The slow growth period was from 2004 to 2014, during which fewer than 100 articles were published each year. From 2015 to 2024, research is growing rapidly, with more than 800 papers published in both 2022 and 2023. In addition, the growth trend in citations is similar to the growth trend in the number of publications. As the number of publications increased, so did the frequency of citations. The increasing trend in the number of publications and citations indicates that gut microbiome-bile acids have been in an active stage in recent years and have received considerable attention from the international community. Moreover, the steady rise in mean citations per publication since 2011 signifies a maturation of the field, where studies are not only accumulating in quantity but also gaining in quality and influence. Given current trends and continued advances in multi-omics approaches and precision medicine, it is reasonable to expect that the number of publications will continue to climb, cementing the gut microbiome-bile acid interaction as a cornerstone of modern biomedical research.

The analysis of countries/regions found that China was the most productive country, followed by the United States. China has become the most productive country, probably because of its increasing investment in scientific research and its large number of researchers. The total link strength (TLS) value is used to assess the level of international cooperation between countries or research groups, and the number of total citations reflects the impact on the field. The United States showed the highest TLS and citations, indicating its active cooperation with other countries and strong international influence. The analysis of institutions showed that 8 of the 10 most productive institutions were Chinese universities. This result is consistent with the fact that China has the highest number of publications (during the current study period). It should be noted that Shanghai Jiaotong University contributed the highest number of publications, and the Chinese Academy of Sciences obtained the largest TLS. These highlight the important influence of China on the study of gut microbiome and bile acids. While China excels in generating a substantial volume of research, the United States may have a more pronounced influence on the global scientific community through its collaborative efforts and the broader reach of its studies. The high frequency of cooperation between China and the United States further emphasizes the importance of their synergistic relationship in advancing the field.

Maps of authors and co-cited authors provide important information about which authors or their teams are more likely to publish more articles or contribute to the work of important information. The h-index is a comprehensive quantitative index used to evaluate the quantity and level of academic output of researchers (Hirsch, 2005). According to our findings, Wei Jia published the most articles, reflecting their substantial research investment, high academic proficiency, and significant contribution to the development of the field of gut microbiome-bile acids. Professor Wei Jia and his team revealed the association of gut microbiome-bile acids with Alzheimers Dement biomarkers (MahmoudianDehkordi et al., 2019; Nho et al., 2019), and their crucial roles in gastric cancer (Jia et al., 2018) and age-related cognitive impairment (Ren et al., 2024). Rob Knight had the highest h-index, indicating that he was a leader in terms of academic influence in the area of gut microbiome-bile acids. Professor Rob Knight’s research uncovered the complex interplay between the gut microbiome and bile acid metabolism, highlighting the significant role of microbiome-derived metabolites in host health and disease, and exploring new methods to improve health and treat diseases by altering the gut microbiome (Fogelson et al., 2023). Jason M. Ridlon was the most co-cited author, indicating that he has played a pioneering role in gut microbiome-bile acids. Professor Ridlon’s research emphasized the role of the gut microbiome in bile acid metabolism, indicating that the gut microbiome is not only involved in the biotransformation of bile acids but also influences the synthesis and transport of bile acids through the farnesoid X receptor (FXR) signaling (Ridlon et al., 2015). These researchers have collectively advanced the understanding of gut microbiome-bile acids interactions, driving the field forward with their impactful studies.

Based on the analysis of journals and co-cited journals, the top five most popular journals were Nutrients, International Journal of Molecular Sciences, Frontiers in Microbiology, Gut Microbes, and Food & Function. As for journal impact, the impact factor (Zimmerman et al., 2022), JCR (Atallah et al., 2020), and h-index (Hirsch, 2005; Engqvist and Frommen, 2008) are potent indicators to value the journals’ impact. Among the top 10 journals, Gut Microbes has the highest impact factor, JCR Q1 journals account for 70%, 5 journals have an H-index greater than 100, and 4 journals have publications more than 100 in research on gut microbiome and bile acids. The underrepresentation of Asian publishers in the top 10 journals, despite substantial contributions from China and Japan, suggests a potential disparity in regional publishing influence within this field.

### 4.2 Knowledge base

A knowledge base represents a compilation of frequently referenced citations within a specific field of study, assisting researchers in mastering the fundamental concepts and principles guiding a new direction of research (Lu et al., 2020). The top-cited articles not only exhibit a high level of academic merit but also exert a substantial professional impact within the domain.

Reference co-citation analysis is a unique analysis method of bibliometrics that is used to study the mutual influence and citation relationship between articles. By analyzing the number of common citations among different references, it can reveal the connections between different research to find out the core references with high influence in this field and present the core hot issues. Notably, among the top ten highly co-cited references, the 2016 publication in Cell Metab stands out prominently (Wahlström et al., 2016). This paper explored the interaction between the gut microbiome and bile acids and their effects on host metabolism. It highlighted that the gut microbiome was not only involved in digestion and nutrient absorption but also influenced host metabolic health by metabolizing bile acids. Bile acids regulated the composition of the gut microbiome and affected host metabolism by activating receptors such as FXR and TGR5. This complex interplay was significant for maintaining host health. These insights provided a scientific basis for the development of new therapeutic strategies, especially in the field of metabolic diseases and gut-related diseases, which increased its importance in medical and clinical research.

The top 10 co-cited references primarily focused on three key aspects: (i) the exploration of the interplay between bile acids, gut microbiome, and host metabolism, highlighting the regulatory role of bile acids in shaping microbiome composition and its impact on metabolic processes; (ii) the investigation of the clinical implications and therapeutic potential derived from understanding the gut microbiome, particularly in the context of obesity and related metabolic disorders; (iii) the identification and analysis of potential targets and mechanisms, including bile acid metabolism, microbiome modulation, and related genetic factors, for advancing gastrointestinal health and disease treatment strategies. Collectively, these three aspects form the cornerstone of research on bile acid-microbiome crosstalk and its applications in improving human health and disease management.

### 4.3 Hotspots, emerging frontiers, and future research directions in gut microbiome-bile acids research

References and keywords analysis can be used to identify hotspots in research fields, which are crucial for predicting potential future directions in a particular field. The top 10 citation references mainly include three aspects. First, the impact of dietary factors on gut microbiome and host health (David et al., 2014; Rowland et al., 2018; Makki et al., 2018). Second, the relationship between gut microbiome and diseases, such as atherosclerosis, rectal cancer, and inflammatory bowel disease (Koeth et al., 2013; Louis et al., 2014; Lloyd-Price et al., 2019). Finally, the interaction between gut microbiome and bile acids and their impact on host health (Tremaroli and Bäckhed, 2012; Wahlström et al., 2016; Sayin et al., 2013; Buffie et al., 2015).

The references with the strongest citation bursts in recent years mainly addressed the significant role of the gut microbiome in human health. Jia W, et al. reviewed the complex interactions between bile acids and the gut microbiome and their impact on the development of gastrointestinal inflammation and cancer, highlighting bile acid metabolism, changes in microbiome composition, and their roles in colorectal and hepatocellular carcinoma by affecting the bile acid-sensitive receptors farnesol X receptor (FXR) and G protein-coupled bile acid receptor 1 (TGR5), it provides a new perspective for understanding the role of bile acid-microbiota axis in gastrointestinal diseases (Jia et al., 2018). Guzior DV, et al. summarized the interaction between human bile acids and gut microbiome, highlighting that gut microbiome modified bile acids and influenced their diversity and functions, with these modified bile acids being associated with various diseases (Guzior and Quinn, 2021). Fan Y, et al. pointed out that the gut microbiome might affect blood glucose regulation, insulin sensitivity, and other aspects, and thus was closely related to the incidence of metabolic diseases such as obesity and type 2 diabetes (Fan and Pedersen, 2021). When the gut microbiome is out of balance, it could lead to the emergence of these diseases. In addition, the study discussed the potential mechanisms by which the gut microbiome influences host metabolism, highlighting the potential application of microbiome-targeted interventions in optimizing metabolic health and preventing disease.

Similarly, the results of keyword analysis reflect the research highlights. According to keywords visualization and keywords citation bursts analysis, metabolic diseases such as obesity, type 2 diabetes mellitus (T2DM), and bariatric surgery are the research hotspots in this field. Obesity is a complex metabolic disease closely related to gut microbiome and bile acids. Studies showed that gut microbiome influenced host metabolism and obesity through multiple pathways that affected gut barrier integrity, production of metabolites and insulin resistance, epigenetic factors, bile acid metabolism, and subsequent changes in metabolic signaling (Lee et al., 2020). Bile acids controlled the overgrowth of gut bacteria, which metabolized bile acids to regulate host metabolism. High-fat diets, disrupted sleep, alcohol, and drug changes in bile acid metabolism reshaped the gut microbiome and contributed to dysbiosis, obesity, and metabolic disorders (Chiang and Ferrell, 2018). Currently, clinical trials of microbiome-based therapies (such as fecal microbiota transplantation and probiotics/symbionics) and BA-based therapies (such as FXR agonists, and TGR5 agonists) are underway as promising therapies for the treatment of obesity-related diseases (Li et al., 2021).

In recent years, with the in-depth study of the relationship between gut microbiome and bile acids metabolism, studies have found that gut microbiome not only plays an important role in the synthesis, biological transformation, and reabsorption of bile acids but also found that gut microbiome-bile acid co-metabolism can act on the metabolism of sugars, lipids and energy of the host (Hou et al., 2023). Studies have found that the imbalance of the gut microbiome leads to decreased production of secondary bile acids and decreased activation of bile acid receptors, which further leads to dysregulation of glucose metabolism and the occurrence of T2DM (Ma et al., 2019). According to various metagenomic studies, it has also been further confirmed that T2DM patients have a significant imbalance in intestinal flora (Lee et al., 2021). The study also found that T2DM was associated with changes in bile acid metabolism, which could be regulated by the gut microbiome. At the same time, bile acids also reshaped the gut microbiome in the bidirectional communication of the gut-liver axis and improved insulin resistance (IR) and T2DM, suggesting that gut microbiome and bile acids may be potential therapeutic targets for T2DM, providing a reference for the discovery and screening of new therapeutic agents (Wu et al., 2020; Gao et al., 2022). Bariatric surgery was originally designed to achieve weight loss and was subsequently found to improve or relieve T2DM. Currently, bariatric surgeries, such as Roux-en-Y gastric bypass and sleeve gastrectomy, are among the most effective treatments for obesity and T2DM worldwide (Liu et al., 2018; Gao et al., 2022).

Meanwhile, mechanism studies such as ‘tgr5’ and ‘pathway’ may be emerging research topics in the near future. Takeda G protein-coupled receptor 5 (TGR5) is a bile acid membrane receptor expressed in various tissue cells, which can sense changes in bile acid concentration and trigger corresponding physiological effects. The study revealed that the gut microbiome indirectly promoted postprandial glucagon-like peptide-1 (GLP-1) secretion by regulating the composition and concentration of bile acids in the ileum, thereby activating the TGR5 receptor on intestinal L cells, suggesting that the gut microbiome could regulate postprandial GLP-1 response through the ileal bile acid-TGR5 signaling pathway (Wang et al., 2023). Castellanos-Jankiewicz et al. (2021) found that hypothalamic TGR5 signaling was a key mediator in the top-down neural mechanism against diet-induced obesity. These newly discovered mechanisms provided important perspectives for understanding the role of gut microbiome in metabolic regulation and also provided potential targets for developing novel therapies against metabolic diseases such as T2DM and obesity.

Based on the above, we summarized and analyzed the hot spots, emerging frontiers, trend analysis, and future research directions of gut microbiome-bile acids, as shown in **Table 7**.

**Table 7:** Research hotspots and future directions in the interaction between gut microbiome and bile acids.

Research hotspots	Trend analysis	Future research fields
Effects of dietary factors on gut microbiome and host health	Dietary intervention studies are gradually increasing, especially focusing on the regulatory mechanisms of specific dietary components (such as prebiotics and dietary fiber) on gut microbiome and bile acids metabolism.	Development of personalized dietary intervention strategies, precise nutrition recommendations based on the characteristics of individual gut microbiome.
Relationship between gut microbiome and disease	The association between gut microbiome imbalance and various diseases (such as atherosclerosis, rectal cancer, inflammatory bowel disease). The research on disease mechanisms has been continuously deepened, shifting from correlation studies to the exploration of causal relationships.	Early diagnostic markers based on gut microbiome of disease development, and targeted treatment strategy of gut microbiome.
Interaction between gut microbiome and bile acids	The interaction between bile acid metabolism and gut microbiome has gradually become a hot topic, especially its role in metabolic diseases.	Targeted therapies for bile acids metabolic pathways, such as FXR and TGR5 agonists, have been developed.
Research on metabolic diseases	Research on metabolic diseases continues to increase, especially the role of gut microbiome in the occurrence and development of diseases.	Intervention strategies for metabolic diseases based on gut microbiome and bile acids metabolism, such as fecal microbiome transplantation and novel drug development.
Bariatric surgery and metabolic improvement	The mechanism of bariatric surgery has been gradually studied, especially its long-term effects on gut microbiome and bile acids metabolism.	Optimized and personalized application of bariatric surgery, and its potential mechanisms in the treatment of metabolic diseases.
Research on mechanism	The research on the mechanism has been deepened, especially the elucidation of signaling pathways and molecular mechanisms.	Development of novel therapeutic targets based on TGR5 and FXR signaling pathways and their application in metabolic diseases.

### 4.4 Limitations

This study provides a systematic analysis of gut microbiome and bile acids using bibliometric techniques, offering insights as a comprehensive guide for scholars interested in this field. However, several potential limitations should be acknowledged in our study. Firstly, the publications’ analysis of gut microbiome and bile acids was only conducted in the past 20 years, and there are some defects in the time of keyword emergence, lacking judgment from the initial stage to the development stage. In the subsequent studies, the statistical start and end time can be extended so that researchers can further study the development history of gut microbiome and bile acids. Second, only the English publications in the Web of Science core collection database were searched for inclusion, while relevant studies published in other languages or indexed in different databases were excluded, which may cause a certain degree of bias in the analysis. Therefore, future research could expand the publication coverage by including additional databases such as PubMed and country-specific repositories to ensure a more comprehensive and nuanced analysis of the field. Finally, data was obtained from bibliometric tools based on machine learning and natural language processing, and particular data processing methods can lead to bias. It is worth noting that bibliometric analysis can only reflect the research status of specific fields to a certain extent and cannot replace traditional reviews. However, compared to traditional reviews, our results are consistent and provide more objective data.

## 5 Conclusions

This bibliometric study revealed that the number of publications in the field of gut microbiome-bile acids research in the past two decades has increased continuously and rapidly, and academic communities around the world are actively collaborating. Among the countries most active in this field, China contributes the largest number of publications, while the United States holds the dominant position in terms of frequency of citations and has a great influence in this field. Based on its high number of publications, large impact factor, and high h-index, *Nutrients*, *Gut Microbes*, and *Journal of Agricultural and Food Chemistry* are currently considered the most influential journals in this field. Jia Wei is the most prolific author, and Jason M. Ridlon is the most frequently co-cited author. Based on the h-index value, Rob Knight and Cani Patrice D. are authoritative and highly co-cited authors. Research hotspots mainly focus on the effects of dietary factors on gut microbiome and host health, the relationship between gut microbiome and a variety of diseases (such as atherosclerosis, rectal cancer, and inflammatory bowel disease), and the interaction mechanism between gut microbiome and bile acids. In recent years, with the development of multi-omics technology, the mechanism of metabolic diseases (such as obesity and type 2 diabetes) has been gradually studied, and the effect of bariatric surgery on gut microbiome and bile acid metabolism has become the focus of research. Future research directions will pay more attention to the development of personalized intervention strategies, the exploration of new therapeutic targets, and the integration of interdisciplinary research, especially the treatment strategies based on bile acid metabolic pathways and signaling pathways (such as TGR5 and FXR), which are expected to provide new ideas and methods for the treatment of metabolic diseases. Our study summarizes the hot spots and future research directions of gut microbiome and bile acids, which can provide valuable inspiration and ideas for further exploration in this field, to promote the development of this field.

## Data Availability

The original contributions presented in the study are included in the article/supplementary material. Further inquiries can be directed to the corresponding authors.
